# Curcumin Attenuates Environment-Derived Osteoarthritis by Sox9/NF-kB Signaling Axis

**DOI:** 10.3390/ijms22147645

**Published:** 2021-07-16

**Authors:** Constanze Buhrmann, Aranka Brockmueller, Anna-Lena Mueller, Parviz Shayan, Mehdi Shakibaei

**Affiliations:** 1 Musculoskeletal Research Group and Tumor Biology, Chair of Vegetative Anatomy, Institute of Anatomy, Faculty of Medicine, Ludwig-Maximilian-University Munich, Pettenkoferstr. 11, D-80336 Munich, Germany; constanze.buhrmann@med.uni-augsburg.de (C.B.); aranka.brockmueller@med.uni-muenchen.de (A.B.); A.Mueller@med.uni-muenchen.de (A.-L.M.); 2 Institute of Anatomy and Cell Biology, Faculty of Medicine, University of Augsburg, Universitaetsstr. 2, D-86159 Augsburg, Germany; 3 Department of Parasitology, Faculty of Veterinary Medicine, University of Tehran, Tehran 141556453, Iran; pshayan@ut.ac.ir

**Keywords:** chondrocytes, curcumin, inflammation, apoptosis, osteoarthritic environment

## Abstract

Inflammation has a fundamental impact on the pathophysiology of osteoarthritis (OA), a common form of degenerative arthritis. It has previously been established that curcumin, a component of turmeric (*Curcuma longa*), has anti-inflammatory properties. This research evaluates the potentials of curcumin on the pathophysiology of OA in vitro. To explore the anti-inflammatory efficacy of curcumin in an inflamed joint, an osteoarthritic environment (OA-EN) model consisting of fibroblasts, T-lymphocytes, 3D-chondrocytes is constructed and co-incubated with TNF-α, antisense oligonucleotides targeting NF-kB (ASO-NF-kB), or an IkB-kinase (IKK) inhibitor (BMS-345541). Our results show that OA-EN, similar to TNF-α, suppresses chondrocyte viability, which is accompanied by a significant decrease in cartilage-specific proteins (collagen II, CSPG, Sox9) and an increase in NF-kB-driven gene proteins participating in inflammation, apoptosis, and breakdown (NF-kB, MMP-9, Cox-2, Caspase-3). Conversely, similar to knockdown of NF-kB at the mRNA level or at the IKK level, curcumin suppresses NF-kB activation, NF-kB-promotes gene proteins derived from the OA-EN, and stimulates collagen II, CSPG, and Sox9 expression. Furthermore, co-immunoprecipitation assay shows that curcumin reduces OA-EN-mediated inflammation and chondrocyte apoptosis, with concomitant chondroprotective effects, due to modulation of Sox-9/NF-kB signaling axis. Finally, curcumin selectively hinders the interaction of p-NF-kB-p65 directly with DNA—this association is disrupted through DTT. These results suggest that curcumin suppresses inflammation in OA-EN via modulating NF-kB-Sox9 coupling and is essential for maintaining homeostasis in OA by balancing chondrocyte survival and inflammatory responses. This may contribute to the alternative treatment of OA with respect to the efficacy of curcumin.

## 1. Introduction

Osteoarthritis (OA) represents the most frequent joint disease that is associated with local inflammation of the synovial membrane, hyaline cartilage tissue, periarticular structures, and subchondral bone, leading to joint pain and physical disability of many millions of people worldwide. This occurs most often after a joint injury from trauma, joint infection, or as a consequence of senescence, wear and tear from the stresses of daily life [1,2,3,4].

Articular cartilage is a hyperspecialized connective tissue with unique biological, mechanical, and metabolic properties that depend on the ultrastructural architecture of the tissue. This connective tissue consists of the highly organized extracellular matrix (ECM), which represents the majority of the tissue, and the resident cells embedded within it, the chondrocytes [5,6,7,8,9]. It is well known that there are very close, specific, and functionally very important interactions between chondrocytes and their pericellular ECM—this interaction is mediated mainly by integrin receptors. It has been more frequently reported that these interactions play a specific role in the process of differentiation and viability of chondrocytes [6,7,9,10,11,12]. Moreover, as a major chondrocyte transcription factor, Sox-9 controls cartilage-concrete ECM formation and is involved in chondrocyte differentiation [13].

Controlled inflammatory processes necessarily take place in our body, for example, as part of the defense against infections. However, if they occur uncontrollably, the inflammation becomes dangerous, injurious, and must be treated. In a joint affected by OA, cytokines like IL-1β, TNF-α, and TNF-β are overproduced by mononuclear cells or synoviocytes. As a result, transcription factors, such as NF-kB and NF-kB-derived proteins, i.e., matrix metalloproteinases (MMPs) and cyclooxygenase-2 (Cox-2), are upregulated, and chondrocytes are unable to compensate for the degraded ECM, leading to morphological and architectural changes, such as cartilage erosion, ECM degradation, and synovial inflammation [14].

Currently, there is no effective pharmacotherapy capable of regenerating the specific structures and functions of the damaged joint and synovial tissue. One of the most important requirements in the treatment of OA is to understand the underlying causes, and thus, find prophylactic and therapeutic ways to alleviate or prevent the main risk factors. The class of substances known as non-steroidal anti-inflammatory drugs (NSAIDs) is used extensively to help relieve inflammation and improve pain in OA sufferers. However, long-term use of NSAIDs has undesirable side effects on many organs, such as the gastrointestinal and cardiovascular systems [15]. However, to date, no effective and healthy therapeutic regimen was created for OA patients. Thus, there is a demand for both safer, more beneficial, and economical new substances for the management of OA [4].

Nature-based compounds have moved into the attention of researchers looking for novel therapeutic ingredients to combat the progression and incidence of OA, as they have the potential for multiple approaches and can help to overcome the disadvantages of symptomatic treatment with many side effects like drug resistance [15]. Medicine has a great history, and so do its natural remedies. Turmeric is a South Asian plant that has long played an important role in traditional Ayurvedic medicine. Its ingredients are also currently the subject of research in all fields. The natural substance curcumin (diferuloylmethane), isolated directly from the rhizome of the healing plant turmeric (Curcuma longa L. Zingiberaceae), is broadly applied in the field of traditional medicine [16,17]. In addition, it has been reported that curcumin has robust potency as an anti-inflammatory, anti-cytokine, anti-bacterial, anti-viral, anti-tumor, cardioprotective agent, and can also suppress cytokine production by specifically suppressing the NF-kB phosphorylation, which is why it is referred to as a natural NF-kB inhibitor [18,19,20,21]. Its modulatory effects on inflammatory processes and related diseases, including OA and RA, have also been studied. In previous studies, our group has demonstrated the anabolic and protective effects of curcumin on IL-1β-, TNF-α-, or TNF-β-stimulated chondrocytes, indicating its chondroprotective properties for the treatment of OA and related osteoarticular problems [22,23,24]. Thus, NF-kB signaling participates in the pathogenesis of OA, has been identified as one of the most important signaling pathways, and significantly regulates OA-associated inflammatory mediators [25].

Here, using an in vivo-like OA-EN model, we demonstrate that curcumin down-modulates the activation of NF-kB and NF-kB-promoted proteins participating in inflammatory and destructive processes of cartilage while upregulating Sox9. These results suggest that curcumin, as a natural multi-targeting component that plays a basal anti-inflammatory role in the inflamed joint, represents a novel therapeutic approach to treat inflammation in OA.

## 2. Results

**Figure 1:**
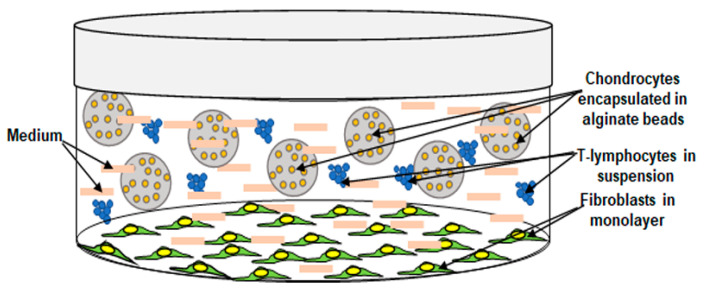
Graphical image showing the basis of the cultural setting of the osteoarthritic environment.

The primary overall concern of this work was to A: Replicate an in vivo-like osteoarthritic environment (OA-EN) in in vitro studies. For this purpose, chondrocytes were cultured as 3D-alginate in pro-inflammatory OA-EN. B: Investigate the specific anti-inflammatory mechanisms of curcumin on 3D-alginate chondrocytes in the OA-EN model in vitro (Figure 1) to provide an in vivo approach OA. This model may help elucidate the role of OA-EN-inducing inflammation and how these actions may be linked by specific signaling pathways during the early onset of human OA.

### 2.1. Curcumin Antagonizes Osteoarthritic Environment-Attenuated Chondrocyte Proliferation, The Same as with NF-kB-ASO or a Targeted IKK Inhibitor

**Figure 2:**
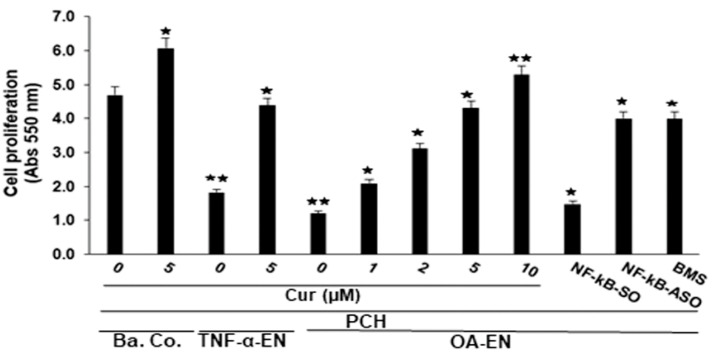
Influence of curcumin, NF-kB-ASO or BMS-345541 on chondrocyte viability and proliferation in the osteoarthritic environment. Chondrocytes in alginate cultures by themselves (Ba. Co.) or in co-culture with fibroblasts and TNF-α (TNF-α-EN) or in co-culture with fibroblasts and T-lymphocytes (OA-EN) were either not treated or treated with different dosages of curcumin (Cur) or with NF-kB-SO/NF-kB-ASO for 14 days, and cell proliferation was monitored with MTT reagents as outlined in Materials and Methods. A minimum of three replicates of each assembly was used. *p* < 0.05 (^★^) and *p* < 0.01 (^★★^) denote a meaningful comparison with the basal control sample as reference.

To investigate the pathological significance of the OA-EN state on chondrocyte viability and proliferation, we first examined the proliferative capacity of chondrocytes in alginate cultures by themselves (Ba. Co.), as well as in co-culture using fibroblasts with TNF-α (TNF-α-EN) or in co-culture with fibroblasts and T-lymphocytes (OA-EN) with and without curcumin treatment as control (Figure 1) and analyzed cell proliferation via MTT method according to the instructions given in the chapter Materials and Methods. To highlight the fact that TNF-α may be considered as one of the key pro-inflammatory cytokines produced by leukocytes, we also performed a comparative experiment with TNF-α alone (TNF-α-EN) in parallel experiments with lymphocytes. As shown in Figure 2, OA-EN with T-lymphocytes or with TNF-α alone significantly inhibited chondrocyte viability and proliferation compared with basal control. Interestingly, in contrast, co-treatment of OA-EN or TNF-α-EN cultures with curcumin showed protective, dose-dependent effects on chondrocytes, resulting in chondrocyte proliferation (Figure 2). Collectively, all these results point to the fact that curcumin indeed has superior anti-inflammatory potency in an OA-EN in vitro. Given that NF-kB is involved in both the inflammatory microenvironment and cellular apoptosis in chondrocytes [26], we further investigated the effect of SO, ASO toward NF-kB or BMS-345541, which is a highly selective IKK component complex blocker and may be capable of specific suppression of NF-kB generation prompted via various types of excitation [27,28] on chondrocyte survival and multiplication via MTT assay, as outlined under Materials and Methods. Treatment of chondrocyte-alginate cultures in the OA-EN with NF-kB-SO significantly downregulated chondrocyte survival and proliferation in a manner similar to the OA-EN itself. Nevertheless, when chondrocyte-alginate cultures were transfected with NF-kB-ASO by itself in the OA-EN, increased proliferation of chondrocytes was seen, resembling that observed in the curcumin-treated OA-EN (Figure 2). Collectively, these lines of available evidence suggest the possibility that TNF-α, analogous to OA-EN, is capable of suppressing chondrocyte survival and proliferation, thus promoting the process by which OA occurs. Curcumin’s specific and targeting suppression of this pro-inflammatory pathway in the OA-EN, similar to the knockdown of NF-kB at the mRNA level by ASO-NF-kB, as well as at the IKK pathway using BMS-345541, is definitely, at least in part, dependent on the NF-kB protein.

### 2.2. Curcumin Antagonizes the Osteoarthritic Environmental-Downregulated sox9 in the Same Way as NF-kB-ASO or a Targeted IKK Inhibitor, as Demonstrated by Immunofluorescence

**Figure 3:**
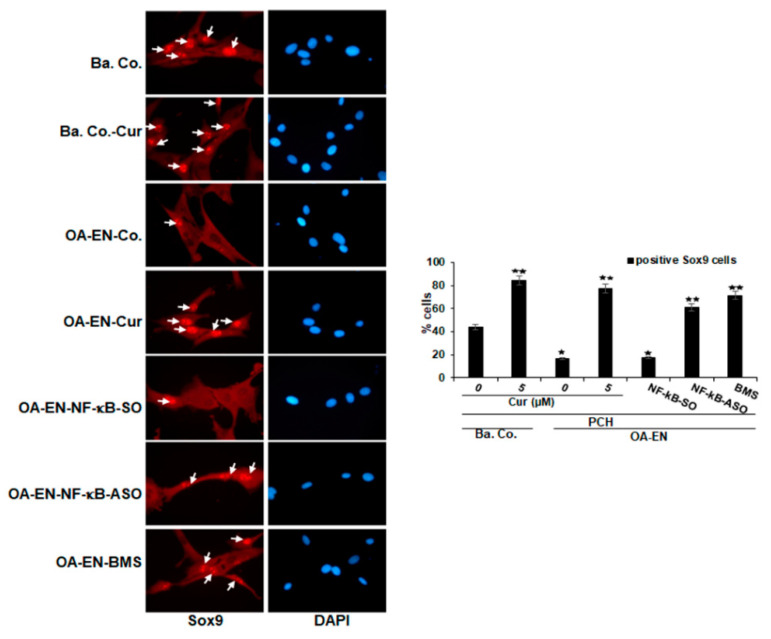
Influence of curcumin, NF-kB-ASO or BMS-345541 upon the Sox9 in OA-EN as visualized by immunofluorescence. Chondrocytes in monolayer cultures by themselves (Ba. Co.) or in co-culture with fibroblasts and T-lymphocytes (OA-EN) were maintained untreated or treated with curcumin for 4 h, or OA-EN cultures were transfected with NF-kB-SO or NF-kB-ASO or treated with BMS-345541 as outlined in Materials and Methods. Figure magnification 600x; scale bar = 30 mm. As a minimum, each experiment was done in triplicate, and positively labeled Sox9 nuclei (white arrows) were quantified based on counting 500–600 cells taken in 25 separate microscopic frames. Scores were matched against control and *p* < 0.05 (^★^), *p* < 0.01 (^★★^) were deemed statistically meaningful.

We next investigated whether the protective and stimulatory effects of curcumin in chondrocytes in OA-EN are mediated by blocking NF-kB and triggering the chondrogenic transcription factor Sox9. Chondrocytes in OA-EN cultures had been handled as outlined under Materials and Methods, following which immunofluorescence staining for Sox9 was done. Basal reference cultures exhibited intensive nuclear fluorescent labeling of Sox9 in chondrocytes (Figure 3), and this labeling was enhanced in the presence of curcumin. In the untreated or in with NF-kB-SO transfected OA-EN cultures, chondrocytes showed markedly lower expression of Sox9. However, pretreatment with curcumin, similar to NF-kB-ASO or BMS-345541, led to a distinct overexpression of Sox9 levels present in chondrocytes (Figure 3). Overall, these outcomes propose that stimulatory effects on specific chondrogenic transcription factor Sox9 by curcumin in the OA-EN, similar to NF-kB-ASO or BMS-345541, are mediated in chondrocytes, which is at least partly through inhibition of the NF-kB regulatory signaling pathway. Furthermore, there is a molecular link between the NF-kB protein and the Sox9 protein.

### 2.3. Curcumin Antagonizes Osteoarthritic Environment-Triggered Reduction of Extracellular Matrix, β1-Integrin, and sox9 in the Same Manner as NF-kB-ASO or a Targeted IKK Inhibitor on Chondrocytes

**Figure 4:**
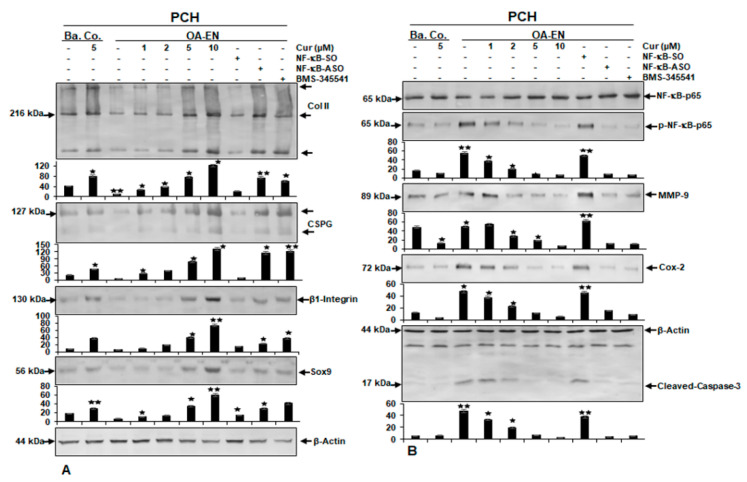
Influence of curcumin, ASO against NF-kB, BMS-345541 upon extracellular matrix, β1-integrin, Sox9, NF-kB, NF-kB-promoted pro-inflammatory, matrix-degrading, and apoptotic proteins on chondrocytes in the osteoarthritic environment. Panel A-B: Serum-depleted chondrocytes (PCH) in alginate cultures by themselves (Ba. Co.) were kept nontreated or treated with curcumin (Cur), or co-cultured with fibroblasts and T-lymphocytes (OA-EN) and kept nontreated or treated with different doses of curcumin, or transfected with NF-kB-SO or NF-kB-ASO or treated with BMS-345541 for 14 days as outlined in Materials and Methods. Total cell samples were obtained, separated via SDS-PAGE, and subjected to western blot assay with antibodies against panel A: Collagen II, CSPG, β1-integrin, Sox9 panel B: NF-kB, phospho-NF-kB, MMP-9, Cox-2, and activated Caspase-3. β-Actin served as a loading control in each assay. Bars represent the mean values for each antibody along with standard variations of at least three separate experiments. Data were compared to the control. Statistically meaningful levels of *p* < 0.05 are marked with (^★^) and *p* < 0.01 with (^★★^).

Previous studies have found that only cells with chondrogenic cell properties can survive in alginate cultures and that alginate acts as a highly specific barrier that isolates healthy chondrocytes from unhealthy chondrocytes and fibroblasts [10,29]. Chondrocytes in alginate from OA-EN cultures in each case have been kept nontreated or handled as outlined in the Materials and Methods chapter. In addition, chondrocytes in the alginate cultures alone served as basal controls. Total cell samples were fragmented and tested by immunoblotting using anti-collagen II, anti-CSPG, anti-β1-integrin, anti-Sox9, and anti-β-actin. As shown in Figure 4, panel A, the pattern of the aforementioned proteins was distinctly reduced in OA-EN compared with basal control cultures. In contrast, treatment of chondrocytes in basal control or in OA-EN cultures with curcumin showed a substantial gain in the expression of aforementioned proteins in a dose-responsive manner (Figure 4, panel A). Next, we aimed to determine whether there was a temporal association of Sox9 with NF-kB signaling pathways. To this end, we used BMS-345541, a potent and highly selective blocker of the IKK protein pathway, or ASO-NF-kB. Interestingly, treatment of chondrocytes in OA-EN with NF-kB-ASO, similar to BMS-345541, significantly increased the pattern of collagen II-, CSPG-, β1-integrin-, and Sox9-expression (Figure 4, panel A), similar to curcumin. In contrast, NF-kB-SO treatment attenuated the levels of protein expression noted earlier in OA-EN cultures, comparable to the control alginate cultures in the OA-EN. Altogether, this implies that the anti-inflammatory benefits of curcumin are partly associated with upstream inhibition of the NF-kB signaling and concomitant promotion to Sox9.

### 2.4. Curcumin Antagonizes Osteoarthritic Environment-Initiated Increase of NF-kB, NF-kB-Promoted Matrix-Degrading, and Apoptotic Proteins in the Same Way as NF-kB-ASO or a Targeted IKK Inhibitor on Chondrocytes

As a means of evaluating that curcumin inhibits OA-EN-induced activation of the pro-inflammatory transcriptional master NF-kB, OA-EN cultures were screened on the activated form of the p65-NF-kB subunit. Based on the findings, the activation of the p65 subunit was significantly increased upon OA-EN cultures compared to basal control chondrocyte-alginate cultures. Curcumin significantly inhibited OA-EN- or basal control-stimulated phosphorylation of p65 subunits in chondrocytes in a dose-dependent fashion (Figure 4, panel B). To further explore the suppressive effects of curcumin targeting in the OA-EN-promoted NF-kB pathway, we investigated upstream OA-EN-triggered IkBα stimulation as a prerequisite to p65-NF-kB activation. Since IkBα phosphorylation and depletion depend on IKK activation, the role of BMS-345541, a directional IKK suppressor, on OA-EN-induced IKK level activity was also investigated. Western blotting results showed that OA-EN induced p65-NF-kB phosphorylation in chondrocytes. The immunoblotting results in Figure 4, panel B show that curcumin possesses an ability with the same intensity as ASO-NF-kB or BMS-345541 to repress p65-NF-kB activity in chondrocytes induced by the OA-EN. Validation of the blots underscores the huge inherent anti-inflammatory capacity of curcumin in the OA-EN, acting selectively on NF-kB. In addition, the expression of NF-kB-triggered proteins implicated in the inflammatory, as well as in destructive events (MMP-9, Cox-2, Caspase-3) was significantly upregulated in the OA-EN cultures compared to basal control chondrocyte-alginate cultures (Figure 4, panel B). Treatment with curcumin in these cultures downregulated levels aforementioned NF-kB-promoted proteins in a dose-dependent way (Figure 4, panel B), similar to ASO-NF-kB or BMS-345541. Densitometric analysis of western blot experiments confirmed the dose-dependent downregulation of NF-kB, MMP-9, Cox-2, and Caspase-3 in chondrocytes in OA-EN cultures treated with curcumin, or with ASO-NF-kB, or with BMS-345541 (Figure 4, panel B). Overall, based on these observations, it appears that the OA-EN promotes chondrocyte degradation, partly through the NF-kB cascade, and that this cascade could be specifically inhibited by curcumin.

### 2.5. Curcumin Promotes the Functional Axis between Sox9 and NF-kB in Chondrocytes in Osteoarthritic Environment

**Figure 5:**
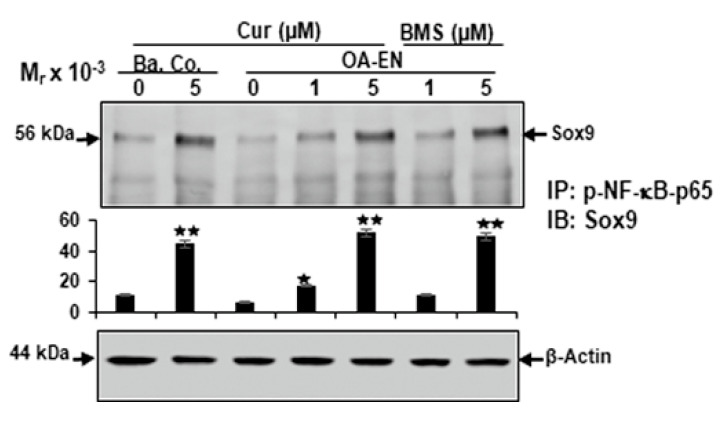
Influence of curcumin, BMS-345541 on the association of p-NF-kB-p65 to Sox9 in chondrocytes grown in OA-EN as shown by co-immunoprecipitation assay. Chondrocytes in alginate cultures by themselves (Ba. Co.) were kept untreated or treated with curcumin or co-cultured with fibroblasts and T-lymphocytes (OA-EN) and either kept untreated or treated with different dosages of curcumin (Cur) or with different dosages of BMS-345541 for 14 days, as outlined in Materials and Methods. Total cell proteins were immunoprecipitated (IP) with p-NF-kB-p65 and optimized by immunoblotting (IB) with anti-Sox9. The initial samples were run using an antibody to β-actin, which acts as a kind of control. Data were compared with the control, and statistically significant meaningful levels of *p* < 0.05 are marked with (^★^) and *p* < 0.01 with (^★★^).

In this report, we showed that NF-kB and NF-kB-stimulated end proteins provoked by OA-EN correlate with a significant reduction of the chondrogenic transcription factor Sox9 and cartilage-specific matrix proteins, demonstrating that both transcription factors act in tandem during OA-EN in chondrocytes. Therefore, to investigate the possible functional association of NF-kB and Sox9 during OA-EN of chondrocytes in alginate cultures, we ran a co-immunoprecipitation assay. OA-EN cultures have been kept untreated or handled as outlined in the Materials and Methods chapter. Additionally, chondrocytes grown solely in the alginate were used as basal controls. Cell samples had been immunoprecipitated using antibodies directed to p65-NF-kB, which was subsequently subjected to immunoblotting by Sox9 antibodies. Interestingly, as shown in Figure 5, in both chondrocytes in basal control and in OA-EN-treated cultures with curcumin, similar to BMS-345541, we found an apparent strong co-immunoprecipitation of p65-NF-kB protein with Sox9 in a dose-dependent fashion (Figure 5). Marginal co-immunoprecipitation of p65-NF-kB protein with Sox9 protein was observed in untreated control cultures (Figure 5). Collectively, this finding further points to the fact that curcumin stimulates the accumulation of Sox9 protein and causes the formation of the Sox9-p65-NF-kB complex in chondrocytes exposed to OA-EN, which activates the chondrogenic differentiation pathway, suggesting that this pathway may be a major part of the anti-osteoarthritic mechanisms of curcumin through Sox9 activation and thereby modulation of the NF-kB pathway.

### 2.6. Curcumin Prevents the Association of NF-kB to DNA in Chondrocytes in Osteoarthritic Environment

**Figure 6:**
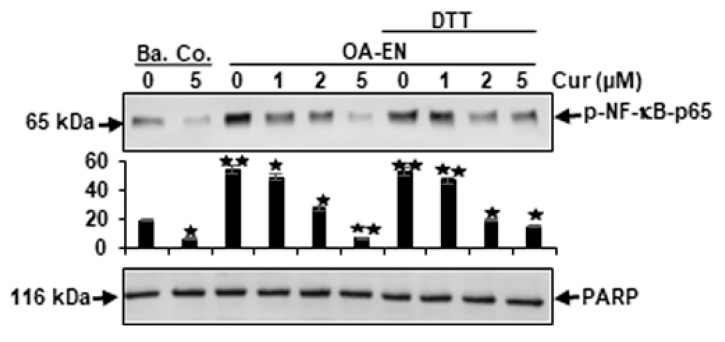
Influence of curcumin on direct association of the p-NF-kB-p65 on DNA in chondrocytes grown in OA-EN. Serum-starved chondrocytes in alginate cultures of OA-EN were handled as outlined in Materials and Methods. Results presented of a minimum of three independent assays, and PARP, a housekeeping protein, was used as a loading control. The densitometric assay was applied to p65-NF-kB. ^★^ *p* < 0.05, ^★★^ *p* < 0.01.

In the past, a number of competing blockers of the p65-NF-kB transcription factor have been frequently published for the binding of p65 to DNA, with the cysteine groups in the p65 subunit being mainly involved in binding to DNA [30,31,32,33,34,35,36]. Therefore, we performed new experiments to see whether reducing cysteine groups with DTT in p65-NF-kB protein affected the association between p65-NF-kB and DNA ligation with and without curcumin treatment as control. Indeed, the results showed that the interference of p65-NF-kB with DNA observed in curcumin-treated chondrocytes, was prevented by DTT in both, basal control and OA-EN cultures (Figure 6). These results show that curcumin markedly inhibits the linkage between p65-NF-kB and DNA, underscoring that this could be one of the primary molecular modes of multi-targeting curcumin by blocking the activation of p65-NF-kB. This section may be divided into subheadings. It should provide a concise and precise description of the experimental results, their interpretation, as well as the experimental conclusions that can be drawn.

## 3. Discussion

To better understand the underlying paracellular interactions between different cells in the pathophysiology of osteoarthritis (OA) and to address the detailed mechanisms during OA in the joint, we created a new osteoarthritic multicellular environment model (Figure 1) consisting of fibroblasts, T-lymphocytes, and 3D-alginate with chondrocytes to better mimic the heterogeneous pro-inflammatory OA-EN in vivo. In the present study, we investigated whether curcumin has the necessary properties to block OA-EN-promoted inflammation in chondrocytes and degradation of ECM by modulating the pro-inflammatory NF-kB transcription factor signaling pathway, as well as the cartilage-specific transcription factor Sox9 signaling pathway, as these are poorly understood.

**Figure 7:**
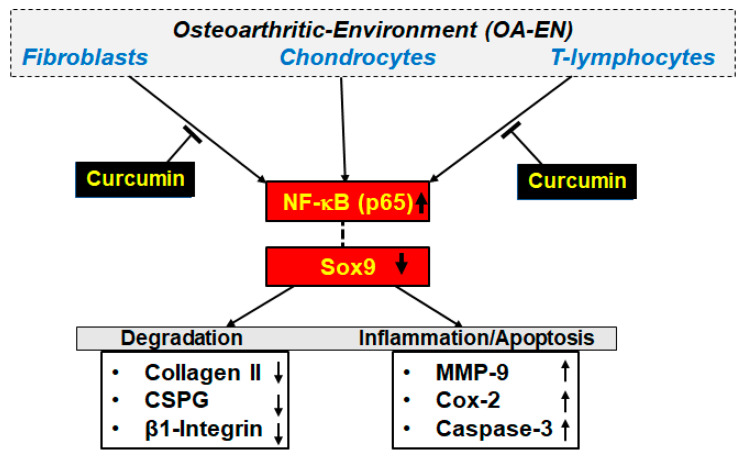
A schematic diagram shows how curcumin modulates the microenvironment leading to osteoarthritis through suppression of inflammatory pathways.

The main findings of the present study were as follows: (I) OA-EN-mediated suppression of chondrocyte proliferation and viability were significantly reduced by curcumin, as well as by knockdown of NF-kB at the mRNA stage using ASO-NF-kB or at the IKK stage using BMS-345541. (II) Curcumin suppressed NF-kB activation induced by OA-EN, as well as inflammatory (Cox-2), ECM-degrading (MMP-9), and apoptotic (cleaved-Caspase-3) proteins controlled through NF-kB (NF-kB-dependent gene products), similar to knockdown of NF-kB with ASO and BMS-345541 in chondrocytes. (III) Curcumin stimulates Sox9 protein and induces the formation of the Sox9-p65-NF-kB complex in OA-EN-treated chondrocytes (Figure 7), activating the chondrogenic differentiation pathway. (IV) We demonstrated for the first time that curcumin markedly inhibited p65-NF-kB activity in the nucleus, which is upregulated by OA-EN, and interestingly, curcumin inhibited these effects in chondrocytes in part by directly inhibiting DNA binding of p65-NF-kB. (V) Finally, curcumin thereby reduced inflammation and chondrocyte apoptosis in OA-EN by positively regulating the expression of Sox9 while inactivating the NF-kB signaling pathway (Figure 7).

OA is associated with inflammation of single or multiple joints in the body and ranges from severe pain to deformities of the joint structures and also of the surrounding tissues [37,38]. Stimulation of multiple interconnected immune-signaling pathways and an imbalance in cytokine production contribute significantly to the pathogenesis and inflammatory response of OA. Therefore, OA therapy using both non-steroidal anti-inflammatory drugs (NSAIDs) and steroids, targets symptoms of the disease, such as relieving pain, and thus, improving joint mobility and disability worldwide [39]. However, these anti-inflammatory drugs only reduce pain and swelling in the joints, but cause severe side effects when taken for prolonged periods. For these reasons, it is also essential to have effective and safe medicines that alleviate OA symptoms in time, or at least delay the progression of the disease [40].

The important results showed that treatment of chondrocytes in OA-EN with curcumin, as well as with ASO against NF-kB or with BMS-345541 (a specific inhibitor of NF-kB activation), inhibited inflammation and chondrocyte apoptosis by suppressing NF-kB signaling. These findings agree with other researchers that the pro-inflammatory NF-kB signaling pathway participating in OA pathogenesis is one of the key inflammatory and catabolic pathways, and also regulates a number of critical inflammatory mediators that are directly associated with the development and progression of OA [23,25,41]. We also found that curcumin strongly inhibited the OA-EN-induced downregulation of chondrocyte proliferation, chondrocyte viability, and the chondrogenic transcription factor Sox9, simultaneously blocked ECM degradation, and chondrocyte apoptosis in the same manner as ASO against p-NF-kB-p65 or BMS-345541. These results clearly indicate that the NF-kB signaling is implicated in the inflammation and cartilage degradation induced by the OA-EN. The NF-kB signaling is one of the major targets of curcumin for its anti-OA effects. We also demonstrated that OA-EN activates the expression of several proteins regulated by NF-kB, including matrix-degrading proteins (MMP-9), as well as the inflammatory enzyme (Cox-2) and the pro-apoptotic protein (Caspase-3), and its stimulation was reversed through curcumin, as well as by ASO against NF-kB or BMS-345541. These outcomes correspond to findings from earlier reports, which reveal that various cytokines or lipopolysaccharides promote activation of NF-kB-induced inflammatory pathway activities and NF-kB-stimulated end proteins participating in inflammation, degradation, and apoptosis in normal or osteoarthritic chondrocytes, and that natural products as multitarget components, such as curcumin and others, can protect chondrocytes and prevent inflammation by specifically reducing NF-kB pathway activation [23,26,27,41,42,43,44,45]. This suggests that a targeted and specific response to the NF-kB signaling pathway could be an essential goal for the inhibition of inflammation, and thus, for the treatment of OA.

Type II collagen and cartilage-specific proteoglycans (CSPG) are key components of the ECM in articular cartilage. Sufficient type II collagen and CSPG are tremendously important for the physiological, biomechanical properties of articular cartilage [46]. It is well known that MMP-9 and Cox-2 enzymes, when activated, are involved in cartilage degradation during OA by degrading and cleaving collagen II and CSPG [47,48]. Furthermore, our results show that curcumin inhibits the expression of MMP-9 and Cox-2 and promotes the expression of collagen II and CSPG in chondrocytes during OA-EN in a manner like the knockdown of NF-kB at the mRNA level by ASO-NF-kB or at the IKK level by BMS-345541. Overall, this set of facts demonstrate that curcumin inhibits the downregulation of ECM production induced by OA-EN by modulating NF-kB activation and NF-kB-terminal proteins in the inflammatory microenvironment, highlighting the protective potential of curcumin in maintaining chondrogenic capacity in chondrocytes.

Interestingly, the NF-kB pathway has previously been reported to be involved in OA pathogenesis by regulating the expression of a number of pro-inflammatory mediators via its phosphorylation [49,50]. It is known that NF-kB initially remains inactive in the cytoplasm under physiological conditions and binds to its inhibitory protein (IkBα). Upon adequate stimulation, e.g., by inflammatory cytokines or the inflammatory microenvironment, phosphorylated NF-kB translocates to the nucleus to associate with DNA and upregulate the expression of inflammation-related genes (such as MMPs, PGE2, Cox-2, iNOS, and NO), promoting ECM degradation in cartilage and chondrocyte death in OA [51,52]. Therefore, targeted modulation of NF-kB activation and NF-kB binding to DNA could contribute much to the treatment of OA.

In this study, we also demonstrated that curcumin inhibited p65-NF-kB binding in the nucleus directly to DNA in chondrocytes, which is upregulated by OA-EN. This result further suggests the other multi-modulatory abilities of curcumin to inhibit inflammation and associated catabolic actions in OA-EN. Furthermore, it has already been reported that the p65-NF-kB subunits are responsible for the interplay of NF-kB and DNA with their cysteine38-group [30]. Moreover, the modulatory effect of this specific interaction by curcumin was restrained with a reducing substance, e.g., DTT, underscoring that curcumin also modulates cysteine groups in the p65-NF-kB unit. These findings are in line with a wide range of supporting research that showed before a number of active ingredients have similar effects to curcumin [53,54,55,56]. These results further demonstrated that curcumin specifically suppressed chondrocyte apoptosis, ECM degradation, and inflammation in OA-EN by specifically modulating NF-kB signaling and NF-kB binding to DNA.

Similarly, we revealed evidence of Sox9, a specific chondrogenic transcription factor, was significantly promoted by curcumin as an indicator of chondrogenic differentiation of chondrocyte activity, like by knockdown of NF-kB at the mRNA level using ASO-NF-kB or at the IKK pathway using BMS-345541. In fact, Sox9 is known to be the most important specific hallmark for chondrogenesis, chondrocyte viability, and survival [57,58]. In addition, we demonstrated for the first time that curcumin can stimulate the expression of the Sox9 protein, while downregulating the expression of NF-kB in OA-EN. Interestingly, the two key transcription factors functionally interacted through curcumin treatment, as shown by co-immunoprecipitation assay, suggesting that curcumin has a modulatory coupling function between inflammation and chondrogenic potential in OA-EN. These results further highlight that curcumin influences maintaining the homeostasis of the balance between chondrogenic potential and inflammatory response, thus modulating the progression of inflammation and chondrocyte degradation in OA in part via the NF-kB-Sox9 axis. As a matter of fact, it was previously published that the regulation of the production of cartilage-specific proteins, such as the cartilage-specific transcription factor Sox9, at both the mRNA and protein levels, is negatively regulated in the OA cartilage by several signaling pathways, and is, thus, associated with high levels of apoptosis [59]. These results are consistent with our findings that curcumin can have a major therapeutic impact on OA-EN by targeting the Sox9 gene via the NF-kB signaling pathway, and, as such, it could have an integral role in the OA pathogenesis.

## 4. Materials and Methods

### 4.1. Materials

Anti-p65-NF-kB, anti-phospho-p65-NF-kB, anti-MMP-9, anti-PARP and anti-activated-Caspase-3 were from R&D Systems (Heidelberg, Germany). Anti-β1-integrin, anti-β-actin, BMS-345541, 4′,6-diamidino-2-phenylindole (DAPI), MTT ((3-(4,5-dimethylthiazol-2-yl)-2,5-diphenyltetrazolium bromide)), dithiothreitol (DTT), curcumin and alginate were from Sigma-Aldrich (Taufkirchen, Germany). Anti-Sox9 was from Acris Antibodies GmbH (Hiddenhausen, Germany) and anti-Cox-2 was from Cayman Chemical (Ann Arbor, MI, USA). Rhodamine-linked secondary antibodies for immunofluorescence were from Dianova (Hamburg, Germany). Sheep anti-mouse/sheep anti-rabbit alkaline phosphatase-associated secondary antibodies for Western blot, anti-CSPG, and anti-collagen type II were from EMD Millipore (Schwalbach, Germany). We prepared curcumin in DMSO (dimethyl sulfoxide) at 5000 µM strain concentration and kept it at −80 °C. For the studies, the final dilutions were carried out in a cell growth medium, and the final DMSO level did not increase above 0.1% during the experiments. Cell growth medium used, was composed of Dulbecco’s modified Eagle’s medium/Ham’s F-12 (1:1) with 10% fetal bovine serum (FBS), 1% glutamine, 1% penicillin/streptomycin dosing (10,000 IU/10,000 IU), 75 μg/mL ascorbic acid, 1% essential amino acids, and 0.5% amphotericin B solution from Seromed (Munich, Germany).

### 4.2. Chondrocyte, T-Lymphocyte, and Fibroblast Culture

We examined primary human chondrocytes supplied by Provitro (Berlin, Germany) as mentioned before [17]. The cartilage specimens of the knee joint (Provitro, Berlin, Germany) were from fully informed patients, and the approval of Charité Universitätsmedizin (Berlin, Germany) had been granted by the medical ethics committee on site. In monolayer growth, chondrocytes were sown at a density of 300,000 cells per T75 cell culture bottle. They grew to a confluency of 70% in a cell growth medium containing 10% FBS. Now the chondrocytes were subjected to up to two passages. Passage 2 and 3 were used in our experiments. MRC-5, a regular human fibroblast cell line, was purchased by the European Collection of Cell Cultures (Salisbury, UK). They also were cultured as monolayer in a T75 cell culture bottle with cell culture growth medium (10% FBS) until 70% confluency was reached, as previously described [60]. We passaged fibroblasts up to two times and used passages 2 and 3 for investigations. Jurkat cells, a human T-lymphocyte cell line [61], were purchased by the Leibniz Institute (DSMZ-German Collection of Microorganisms and Cell Cultures, Braunschweig, Germany). They served as cultures in suspension with a total culture growth medium supplemented with 10% FBS. We cultured and maintained cell lines under standard culture settings at 37 °C and 5% CO_2_. Before initiating an experiment, cells underwent three washes with serum-starved medium (3% FBS) and were incubated in the unchanged medium for 30 min.

### 4.3. Chondrocyte Transfection

The transfection of chondrocytes was carried out as before mentioned [62]. Phosphorothioate-specific oligonucleotides (21-mer) from Eurofins MWG Operon (Ebersberg, Germany) were used to perform transient transfection with antisense/sense (ASO/SO) oligonucleotides based on NF-kB. ASO order was 5′-gGAGATGCGCACTGTCCCTGGTC-3′, corresponded to NF-kB/p65 subunit mRNA, and SO control order was 5′-gACCAGGGACAGTGCGCATCTC-3′. To keep the oligonucleotides safe from cell nucleases, ASO and SO were phosphonothioate-changed. Before starting the experiments, chondrocytes were transfected by incubating the cells as confluent monolayers for 24 h using 0.5 µM NF-kB-ASO or NF-kB-SO and 10 µL/mL Lipofectin (Invitrogen, Karlsruhe, Germany) in the serum-depleted medium.

### 4.4. Osteoarthritis Model In Vitro

The objective of the present research was to construct a 3-dimensional (3D) osteoarthritic environment (OA-EN) model in vitro to mimic an in vivo joint-like OA-EN to examine the impact of curcumin in the modulatory intercross talk among chondrocytes and their microenvironment (Figure 1). As “basal control (Ba. Co.)”, chondrocytes were encapsulated in alginate beads, were cultured with or without 5 µM curcumin treatment as a control in whole-cell culture medium. To accomplish the “OA-EN”, fibroblasts cells were seeded as a monolayer (3.000/cm^2^) in Petri dishes and incubated for 24 h in whole-cell culture medium with 10% FBS as it was precisely explained before [53]. Now, chondrocytes were encapsulated in 3D-alginate beads as presented before [10,27,29], and finally, 3D-chondrocyte-alginate beads were co-cultured with 10.000 Jurkat cells/mL (T-lymphocytes) in Petri dishes containing the fibroblasts in serum-starved medium (3% FCS). This composition of chondrocytes 3D-alginate beads, Jurkat cells, and fibroblasts serves as the “OA-EN-Co.” model in vitro. As a comparison, we simulated the OA-EN without T-lymphocytes, but with pro-inflammatory cytokines, TNF-α. Cultures of chondrocytes in alginate matrix alone (Ba. Co.) or in co-culture with fibroblasts and TNF-α (10 ng/mL) or in the OA-EN model were (1) untreated or (2) treated with different doses of curcumin (1, 2, 5, 10 μM) or (3) transfected with 0. 5 μM SO/ASO against NF-kB in the presence of Lipofectin (10 μL/mL) or (4) treated with BMS-345541 (5µM) for 10 days.

### 4.5. Vitality Assay

To evaluate the proliferation potential and viability of chondrocytes in OA-EN in the presence or absence of curcumin, cells were first examined in 3D-alginate cultures by themselves (Ba. Co.) or in co-culture with fibroblasts and TNF-α (TNF-α-EN) or in co-culture with fibroblasts and T-lymphocytes (OA-EN) with and without curcumin treatment as control (Figure 1). After 10 days in culture, cells were first detached from the alginate matrix, and then an MTT assay was performed as already reported [27,53,63]. For this, the alginate beads were dissolved in sterile 55 mM sodium citrate solution for 20 min to free the chondrocytes from alginate. Now the cells were washed with Hanks saline, resuspended in a modified cell culture medium (3% FBS, without phenol red, without vitamin C), and spread on a 96-well plate with 100 µL cell suspension and 10 μL MTT solution (5 mg/mL) on each well. The reaction was stopped by adding 100 µL MTT solution (10% Triton x-100/acidic isopropanol) to each well after 3 h. The plate was kept at 37 °C overnight. The next day, optical density (OD) was determined at 550 nm (OD550) using a Revelation 96-well multi scanner plate ELISA reader from Bio-Rad (Munich, Germany).

### 4.6. Immunofluorescence Investigation

The intranuclear localization of the cartilage-specific transcription factor Sox9 protein, due to the above-described treatment in osteoarthritic settings was investigated by immunofluorescence, as precisely outlined earlier [17,27,63]. Chondrocytes were grown as a monolayer with 5000 cells/glass plate. The next day, glass plates were placed on small steel grid bridges in Petri dishes with fibroblasts at the bottom, and 10,000 T-lymphocytes per ml of cell culture medium were added before treatment (5 µM curcumin, 5 µM BMS-345541, 0.5 µM NF-B-ASO or NF-B-SO) began. After 4 h of treatment, glass plates were fixed with methanol for 15 min, washed with PBS/BSA (1%), and incubated overnight with primary antibodies in a humidity chamber at 4 °C. The next day, cells were incubated with secondary rhodamine-coupled antibodies for 2 h and 15 min with DAPI to stain the nuclei and were then embedded in Fluoromount from Sigma-Alderich (Munich, Germany).

### 4.7. Western Blot Analysis and Immunoprecipitation

Western immunoblotting of chondrocytes from alginate cultures of various osteoarthritis cultures was performed with whole-cell lysates, as reported in depth in our former papers [17,23,26,44,60]. Immunoprecipitation assays were performed to investigate the important functional relationship between NF-kB protein and endogenous signaling interactions with Sox9, as this technique has been demonstrated in depth before [6,10,27,63]. In short, the chondrocytes were trypsinized and washed in ice-cold PBS. Now the cell pellets were resuspended in hypotonic lysis buffer and incubated for 15 min. Samples were reduced with 2-mercaptoethanol. Proteins were separated by SDS-PAGE. We used a transblot apparatus from Bio-Rad (Munich, Germany). After preincubation in blocking buffer (PBS, 5% skim milk powder, 0.1% Tween 20), we incubated the membranes overnight with primary antibodies and for 90 min with secondary antibodies. Antigen-antibody complexes were detected with nitroblue tetrazolium and 5-bromo-4-chloro-3-indoyl phosphate from VWR (Munich, Germany). Bindings were quantified by densitometry using the “Quantity One” program from Bio-Rad (Munich, Germany). To investigate the relationship between NF-kB protein and endogenous signaling interactions with Sox9, immunoprecipitation assays were performed as previously described [27]. Cell extracts were pre-cleared by incubation with 25 µL of normal rabbit or mouse IgG serum and Staphylococcus aureus (SAC), then incubated with primary antibodies at 4 °C for 2 h, followed by incubation with SAC at 4 °C for 1 h. Samples were separated by SDS-PAGE. 

### 4.8. DNA Binding Assay

We conducted a DNA binding assay to see whether reducing cysteine residues by DTT in NF-kB protein could alter the association of p65-NF-kB with DNA binding with and without curcumin treatment as control, as presented in depth in earlier work [18,53,56]. In brief, chondrocytes were cultured in alginate OA-EN for 10 days. Cytoplasm extraction buffer was used to extract their cytoplasm. Nuclei were left untreated or treated with curcumin (1, 2, 5 µM) and DTT (5 mM) in combination or alone for 1 h. After preparing nuclear extracts, proteins were separated for immunoblotting (SDS-PAGE) and analyzed for NF-kB activation.

### 4.9. Statistical Evaluation

All in vitro tests were carried out as single assays three times with three replicates each. A Wilcoxon-Mann-Whitney test was used for statistical calculation. All data were presented as mean + SD or SEM and related by one-, two-, or three-way ANOVA using SPSS statistics if the test for normality (Kolmogorov-Smirnov test) was successful and a *p*-value < 0.05 indicated statistically meaningful deviations.

## 5. Conclusions

The present study has shown that curcumin can reduce OA-EN-induced inflammation, consequently, ECM degradation and chondrocyte apoptosis by targeting the NF-kB-Sox9 signaling axis (Figure 7). These findings were confirmed by the same results obtained by treating the same cultures with ASO-NF-kB or with BMS-345541. Our results showed that: (1) Curcumin attenuates the effect of OA-EN-related catabolic factors, (2) curcumin functionally couples inflammation and chondrocyte assembly through positively regulating the expression of Sox9, while inactivating the NF-kB signaling pathway, (3) this highlights the maintenance of homeostasis that balances chondrocyte survival and inflammatory responses to prevent the occurrence of OA or attenuate OA that has already occurred. These outcomes highlight the inherent multitargeting polyphenolic value of curcumin in clinical treatment in OA in the future.

## Data Availability

All data are available in the manuscript.

## Abbreviations

ASO-NF-kB	Antisense oligonucleotides targeting NF-kB
Caspase-3	Cysteine-aspartic protease 3
COX-2	Cyclooxygenase
CSPG	Chondroitin sulfate proteoglycan
DTT	Dithiothreitol
EN	Environment
IKK	IkB kinase
NF-kB	Nuclear factor kappa B
MMP-9	Matrix metalloproteinase 9
mRNA	Messenger RNA
OA	Osteoarthritis
Sox-9	SRY-Box Transcription Factor 9
TNF-α	Tumor necrosis factor alpha
